# Ethnopharmaceutical knowledge in Samogitia region of Lithuania: where old traditions overlap with modern medicine

**DOI:** 10.1186/s13002-018-0268-x

**Published:** 2018-11-20

**Authors:** Zivile Pranskuniene, Roberta Dauliute, Andrius Pranskunas, Jurga Bernatoniene

**Affiliations:** 1 Department of Drug Technology and Social Pharmacy, Lithuanian University of Health Sciences, Sukileliu pr. 13, Kaunas, Lithuania; 2 Institute of Pharmaceutical Technologies, Lithuanian University of Health Sciences, Kaunas, Lithuania; 3 Department of Intensive Care, Lithuanian University of Health Sciences, Kaunas, Lithuania

**Keywords:** Ethnopharmacy, Samogitia, Urban ethnobotany

## Abstract

**Background:**

Modern ethnopharmaceutical studies are still quite unusual in Northern Europe. Data regarding the medicinal use of plants, animals, and fungi and also of spiritual rituals of healing is obtained mostly from ethnographic and folkloric sources in Lithuania. The aim of this study was to assess the ethnopharmaceutical knowledge regarding traditional use of natural substances for medicinal purposes in the Samogitia region and compare with prior research conducted 10 years prior in the same region.

**Methods:**

The study was performed during 2016–2017 in the Samogitia region (Lithuania) using the conventional technique of ethnobotanical studies. Twenty-eight respondents aged between 50 and 92 years were selected for the study using snowball techniques. Information was collected using semi-structured and structured interviews. The obtained information was recorded indicating local names of plants, their preparation techniques, parts used, modes of administration, and application for therapeutic purposes.

**Results:**

During the research, 125 records of raw materials of herbal origin belonging to 55 families were made. The Asteraceae family had the highest number of references, 147 (16.6%). It was stated that the most commonly used medicinal plants were the raspberry (*Rubus idaeus* L.) (100%), marigold (*Calendula officinalis* L.) (96.4%), camomile (*Matricaria recutita* L.) (92.9%), and small linden tree (*Tilia cordata* Mill.) (92.9%). The most commonly used material of animal origin was the toad (*Bufo bufo*) (89%). The most commonly used kind of fungi was the common stinkhorn (*Phallus impudicus*) (71%), and the material of the mineral origin was sand (50%). Comparative analysis of the two surveys in this region showed similar results and produced a large amount of ethnopharmaceutical information.

**Conclusions:**

Lithuania belongs to the countries known for urban ethnobotany where old traditions overlap with modern healing methods. Also, because modern medical assistance is quite expensive, self-medication with home-made medicines is still popular in Lithuania. It is important to collect and systematize this information as soon as possible, to save it as a traditional Lithuanian heritage and also use it for scientific investigations.

## Background

Despite the growing number of ethnopharmaceutical studies in Europe [1–5], publications in Lithuania are scarce [6]. Scientists from Poland, a neighboring country sharing historical and cultural background in this study field, wonder why a country with old folk medicine traditions and a generous folk medicine archive would not attempt to reach a wider audience. In contrast to some other Eastern European countries such as Poland, Hungary, or Estonia [7, 8], there are very few publications concerning the ethnobotany of Lithuania [6, 9]. A review of medico-ethnobotanical field studies in Europe [1, 3, 7] shows that Lithuanian ethnomedicinal studies have made a very small contribution to European ethnomedicinal studies, being mainly done by researchers of neighboring countries, which were a part of historical regions of Lithuania. Modern medico-ethnobotanical studies are still quite rare in Northern Europe, and data regarding the use of plants and people’s perceptions of plants has mostly been studied based on ethnographic and folkloric sources of Lithuania [9]. In various historical periods, ethnomedicinal expeditions were organized with the aim of preserving local knowledge about traditional uses of medicinal plants, animals, and fungi and also spiritual rituals of healing. All these findings were mostly collected in the Lithuanian language, often in local community’s dialects, and stored in the archives [10]. The problem about this information is methodological guidelines. Usually, this information was collected as an additional file, while the main part of research was focused on folkloric-ethnographic studies [11, 12].

Samogitia is a region with a unique history in Lithuania. In 1417, after the baptism of Samogitians, the bishopric of Samogitians was founded in Varniai, one of the spiritual and cultural centers of Lithuania [13]. Catholic monasteries played a paramount part in spreading phytotherapeutic practice [14]. The priest, a botanist and Samogitian folk physician Pabrėža J., tried to apply his own knowledge to medicine, to make medicines, to educate people about herbs and encourage their use for treatment, to visit patients, and to acquiesce and treat them in the monastery. Also, in 1814, he wrote a treatise on the medicinal herbs common in Lithuania, their medicinal properties, the prevailing recipes, and the ways of producing the necessary medicinal products from the medicinal plant material [15]. According to Kujawska et al. [7], opportunities to practice traditional forms of healing were probably also more favorable in Lithuania in comparison with Western Europe or neighboring country Poland.

Lithuania belongs to the countries of urban ethnobotany, where old traditions overlap with modern healing methods. From the point of view of ethnologists, urban culture started taking over Lithuanian villages in the seventies of the twentieth century. On the other hand, according to reports of tsarist Russia doctors, even by the end of the nineteenth century, most of the population had had very little encounter with biomedicine. Polish researchers of that time had also pointed out that in Lithuania, the oldest elements of traditional medicine had survived [16]. Data of the end of the twentieth century note at least 500 plant species have been utilized as traditional remedies [14]. According to the latest data, 462 spontaneous, adventitious, or introduced species of higher plants, five species of mushrooms, two of lichens, one of moss, and one of algae are still used in traditional and Lithuanian folk medicine [17].

The earlier studies done in this field [6] and analysis of archival sources [10] have discovered a big amount of information about traditionally used plant-, animal-, and mineral-based medicines and even ritual healing procedures.

Ten years ago, there was an urban ethnobotany study done in this region. It was the first pilot study in Samogitia (Lithuania), which presented a survey on the preserved knowledge of the local population about the usage of medicinal plants for therapeutic purposes and on its applicability in modern primary healthcare [6]. Our ancestors used all nature’s gifts for medical treatment: medicinal plants, fungi, animals, and mineral substances. According to Lithuanian ethnographers, the Lithuanian folk medicine is conditionally divided into rational and magical methods of treatment [15]. Researchers from neighboring countries argue that ethnomedicine in Lithuania may be lacking in scientifical investigation because the main focus so far has been made on verbal formulas, associated with charm healing, and that was the cause of a stereotypical perception of traditional medicine as an irrational practice [7]. As a result, during the study, an effort was made to capture all the means, suppositions, incitements, or spells used for the treatment, as all this is trend of gradually disappearing Lithuanian folk medicine. This study tried to encompass all kinds of materials used for medicinal purposes because our earlier study of archival sources showed a wide use of remedies of animal origin [9].

We hypothesized that despite positive changes in modern medical assistance during 10 years, and despite increased accessibility to commercially produced remedies that can be purchased at pharmacies or recommended by qualified physicians, local inhabitants in villages still actively use traditional medicine.

The main tasks of the research were as follows: (1) to assess the source of ethnopharmaceutical information, (2) to evaluate how many respondents choose their healthcare professional (pharmacist or doctor) as a qualified consultant regarding herbal remedies, and (3) to compare results with prior research 10 years ago in the same region by identifying the most commonly used medicinal plants, animals, and fungi species.

This study contributes to the ethnomedicine database with collected material, which will be left in the Lithuanian Museum of the History of Medicine and Pharmacy and used for educational purposes for healthcare students. Also, presenting this study to the scientific community will fill the gap in the field of ethnomedicinal studies in the context of modern Europe.

## Materials and methods

### Study area

Lithuania is situated in the northern part of the middle latitude climate. It means that the biggest influence on the climate in Lithuania has the Atlantic Ocean, especially the Gulf Stream, which washes West European shores. Usually, Lithuania has medium range air masses (80–87%): continental air masses are more frequent in spring and summer and maritime in autumn and winter. The average annual temperature in Lithuania is 6.2 °C, and the difference between the warmest month (July) and the coldest month (January) can reach 21.8 °C; the average annual precipitation is about 550–850 mm [18].

In Lithuania, native tree species prevail: a share of coniferous tree species in the overall species composition is 58.2% with Scots pine (*Pinus sylvestris*) (36.2%) and Norway spruce (*Picea abies*) (21.8%) making up the biggest part. Deciduous trees are mainly represented by birch (20.6%), black alder (6.6%), and gray alder (6.3%) [18].

**Fig. 1:**
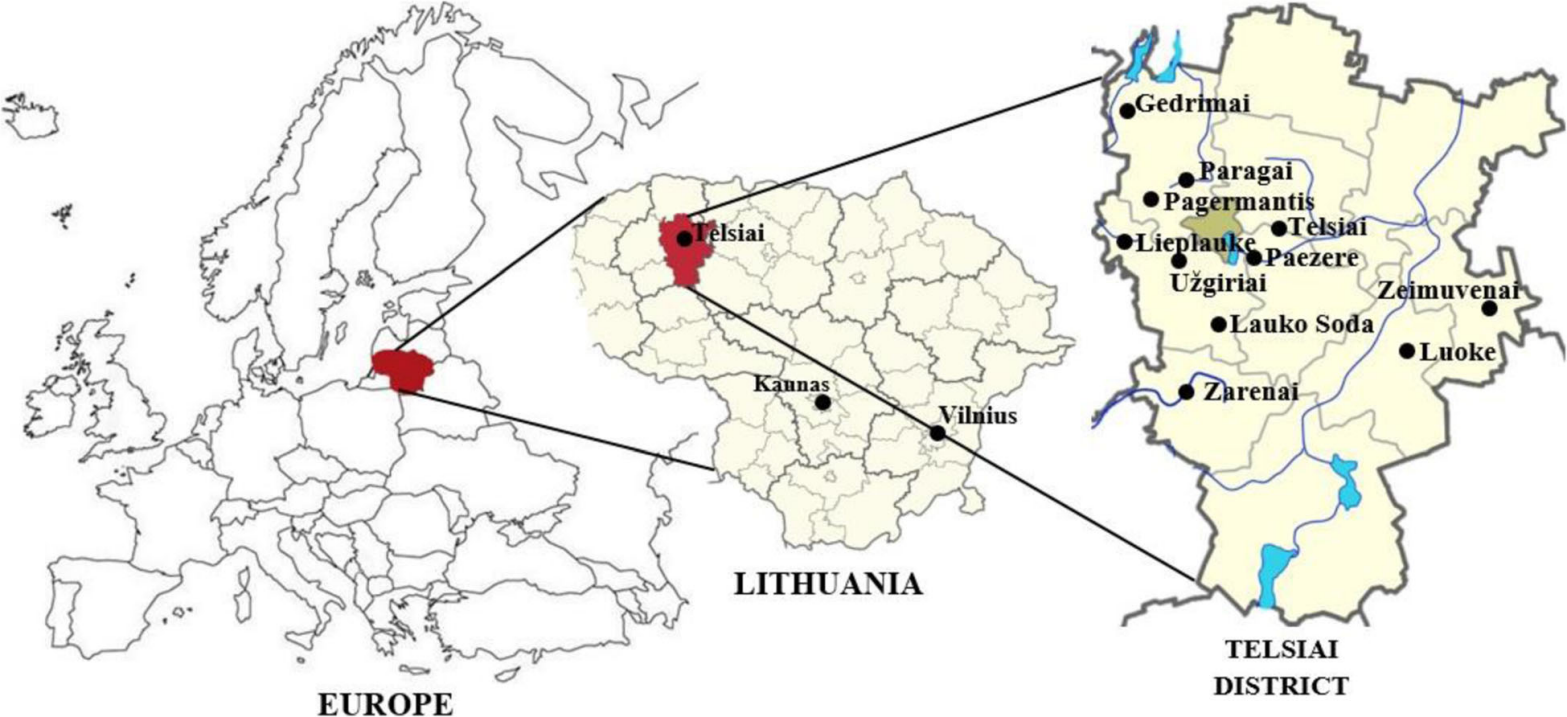
Study area

The study was performed in the central part of the Samogitia region located in the western part of the country, Telšiai County (Fig. 1). This region has preserved Samogitian language (a dialect, quite different from the standard Lithuanian language), manners, and old traditions. A small rivulet, the Telšė, which flows into the lake Mastis, gave its name to Telšiai. The town was first mentioned in written sources around 1450, but the earliest archeological findings in the area of the town are from the Stone Age. For the first time, Telšiai County (as the Telšiai repartition) was formed in 1764. In 1935, Telšiai became the center of county administration. In 2016, the population in Telšiai County was about 139,000 [19].

The main location of the study was Telšiai district, especially its eastern part. The district is located in the hilly Samogitian highlands. The northern part of it belongs to Samogitia National Park, the southern to Varniai Regional Park. There are numerous mounds, ancient burial grounds, mythological and sacred stones, and natural objects. This area is distinguished by its forests and peat bogs. The forest area of Telšiai district is 30.9%, mostly spruce, pine, and mixed forests. There are 50 lakes, six ponds, and several rivers flowing through the district [19].

### Methods

Field work was conducted during the periods of June–August 2016 and April–August 2017. The purpose of the study was explained to every person, and prior informed consent was obtained from all interviewees. We followed the Code of Ethics of the International Society of Ethnobiology [20]. The research was approved by the Bioethics Center of Lithuanian University of Health Sciences (No. BEC-FF-19).

In the initial stage of field work, information about the area of the study was collected, and we were acquainted with the locality, people, and a culture of communication in the region. Approval to carry out this type of research was obtained from the local community and with local authorities, and a permit for collecting material was obtained. Ethnobotanical information was collected using semi-structured and structured interviews with 28 persons. Twenty-four women and four men aged between 50 and 92 years were interviewed in depth about ethnomedicinal preparations. Nowadays, in Lithuania, women live 10.5 years longer than men, and after 50 years, the number of men is significantly decreasing; therefore, the percentage of men and women is uneven [21]. However, the main reason why so few men participated in the survey is the fact that traditionally, folk medicine in Lithuania was transmitted through the women’s line, and only in the absence of a daughter in family, then were trusted to a male descendant with proper characteristics [12]. The interviewed people were mainly herbalists, farmers, and housewives, who still had strong connections with traditional agricultural activities. Study guides selected these persons as users of traditional medicine. The study sample was selected using a snowball technique—the interviewed persons recommended other individuals. The first meeting with the interviewed persons occurred at their respective homes, in their gardens, and fields. Many of our respondents lived in lonely farmsteads with the nearest farmstead or settlement a few kilometers away. The respondents were mostly farmers, housewives, and herbalists. Only few respondents worked in town but still lived in farmsteads growing various plants for their own needs. The Samogitians are quite modest and reserved people. At first, they are not very willing to speak, so it takes some time to get the respondents to relax. The Samogitians are proud of their traditions, and even today, they are widely using their traditional medicine [19].

The study consists of two stages. First, by using the completed questionnaire, an interview was conducted during which the investigator himself wrote down the answers to the questions. The questionnaire consists of 13 questions: main questions for demographic data and also closed questions to assess the source of ethnopharmaceutical information and to evaluate how many respondents choose a healthcare professional (pharmacist or doctor) as a qualified consultant regarding herbal remedies. The main focus was on the conditions for collecting and storing home-made medicinal products, how many products they already have at the time of the interview, and what kind of medicinal herbs they cultivated.

The second step was to obtain as much information as possible by recording local names of plants, their preparation techniques, parts used, modes of administration and application for therapeutic purposes, use of single or mixture of plants for remedy preparation, dose requirement, and usable duration regarding each medicine. In addition to documenting current uses, we also asked interviewees to recall the uses of plants they had used themselves or observed their parents using in the past. Information concerning other traditional remedies used in local folk medical practices was also collected. Materials of animal, mineral, and other origin were considered. This information was used in the form of a free interview, and informants were allowed to speak spontaneously and without pressure. Our final purpose was to obtain a complete list of medicinal plants used and/or known by each informant. In the course of the study, it was observed that anonymity and some additional visits to participants led the interviewees be more inclined to communicate, revealing their traditional methods of treatment, prescriptions, and incitements which were important or even kept secret. The fact that participants did not need to answer questions in writing, that questions were simple and clear, and that the survey was a free form of communication, all contributed to the active participation of the respondents.

Interviews were voice recorded with permission from the interviewee, and field notes were also taken and encoded. During the study, it was attempted to capture information about collected medicinal substances: where they were collected and how and under what conditions they were dried and stored. Parts of plants were identified using writings on traditional Lithuanian flora [22–24]. The persons were asked to recognize all the used species by pictures and in vivo plants. Also, when possible, they were asked to supply a fresh sample of each plant mentioned for the taxonomic determination and name it in local dialect. Folk names of local plants were identified using the rules of Samogitian dialect and linguistic methodology of Lithuanian ethnobotanical taxa [25]. Whenever possible, plant voucher specimens were taken or plants identified on the basis of dried samples. As it was not the full vegetation season, some plants were identified on the basis of their vernacular names and a full description provided by the interviewee. In the cases of interviewees treating a genus as one unit, the plant was identified at the genus level only. Plant samples were collected from the field and were assigned voucher numbers and deposited at Lithuanian Museum of the History of Medicine and Pharmacy *Materia medica* section. Taxonomic identification, botanical nomenclature, and family assignments followed the Plant List database [26] and the Angiosperm Phylogeny Group IV [27]. The frequency of reports indicates the number of informants who mentioned the use of each species. Because several medicinal uses are often mentioned by different informants, the frequency of reports for each specific illness of every plant is of high value (Table 1).

**Table 1:** Herbal material used for medicinal purposes

Family	Botanical name, voucher no.	Disorder	Frequency of citations	Part used	Preparation	Administration and dosage
Acoraceae	*Acorus calamus* L.	Indigestion and appetite loss (C)	1	Roots	Tea	O. Ad.
		Hair loss (C)	1		Decoction	Ext.
		Constipation (C)	2		Tea	O. Ad.
Adoxaceae	*Sambucus nigra* L. (MFMTR20)	Kidney and urinary tract diseases (C)	2	Flowers, fruits, bark	Tea	O. Ad.
		Respiratory tract disorders (C)	3			Inhalation
Amaryllidaceae	*Allium sativum* L.	Toothache (C)	2	Corms	Put on an aching tooth	Ext.
		Cold, respiratory tract disorders (C)	8		Eat corms, put into the nose, rub the neck and chest	O. Ad., Ext.
		Vermin diseases (C)	2		Eat corms, drink juice	O. Ad.
	*Allium cepa* L.	Cold, respiratory tract disorders (C)	7	Corms	Cut, boil in milk, put honey	O. Ad., one teaspoon every hour, few days
		Indigestion and appetite loss (C)	3		Cut, mixed with honey	O. Ad., one teaspoon before meal
Apiaceae	*Aegopodium podagraria* L. (MFMTR01)	Gout (C)	2	Flowers, leaves, roots	Tea, decoction	O. Ad.
		Kidney and urinary tract diseases (P)	1		Tea, decoction	O. Ad.
		Joint pain (C)	1		Compress	Ext.
	*Levisticum officinale* W. D. J. Koch. (MFMTR11)	Gout (C)	2	Roots	Decoction	O. Ad.
		Indigestion (C)	1		Decoction	O. Ad., 30 min before meal
		Male potency disorders (C)	1		Decoction	O. Ad.
		Kidney diseases (P)	1		Decoction	O. Ad.
	*Anethum graveolens* L. (MFMTR16)	Indigestion and appetite loss (C)	3	Fruits	Decoction	O. Ad., 30 min before meal
		Inflammatory diseases (P)	1		Decoction	O. Ad.
	*Daucus carota* L.	Cardiovascular system disorders (P)	2	Leaves	Tea	O. Ad.
		Constipation (C)	4	Roots	Juice	O. Ad.
		Immunodeficiency (C)	3		Juice	O. Ad.
	*Foeniculum vulgare* Mill.	Gastric and intestine diseases (C)	1	Fruits, seeds	Extract with alcohol	O. Ad.
		Respiratory tract disorders (C)	1		Extract with alcohol	O. Ad.
	*Apium graveolens* L.	Constipation (C)	1	Roots	Extract with alcohol	O. Ad.
		Kidney and urinary tract diseases (P)	1		Extract with alcohol	O. Ad.
	*Carum carvi* L.	Indigestion and appetite loss (C)	7	Seeds	Extract with alcohol, decoction	O. Ad.
		Constipation (C)	3		Decoction	O. Ad.
	*Petroselinum crispum* Mill.	Indigestion and appetite loss (C)	4	Aerial parts	Decoction	O. Ad.
		Depletion (C)	2			O. Ad.
Asparagaceae	*Polygonatum odoratum* (Mill.) Druce. (MFMTR18)	Inflammatory diseases (P)	2	Roots, herb	Extract with alcohol, decoction	O. Ad.
Asphodelaceae	*Aloe vera* (L.) Burm. f.	Skin diseases (C)	4	Leaves	Juice	Ext., rinsing
		Indigestion and appetite loss (C)	2			O. Ad.
Asteraceae	*Tanacetum vulgare* L. (MFMTR31)	Vermin diseases (P)	2	Flowers	Decoction	O. Ad.
		Cold, fever (C)	1		Decoction	O. Ad.
	*Inula helenium* L. (MFMTR35)	Respiratory tract disorders (C)	1	Roots	Decoction	O. Ad.
		Kidney and urinary tract diseases (C)	1		Decoction	O. Ad.
	*Bidens tripartita* L. (MFMTR55)	Allergies (P)	1	Aerial parts	Decoction	O. Ad.
		Purulent wounds (C)	1		Decoction	Ext.
	*Echinacea purpurea* (L.) Moench.	Cold, influenza (C)	2	Aerial parts, roots	Extract with alcohol	O. Ad.
		Respiratory tract disorders (C)	2		Extract with alcohol	O. Ad.
		Skin diseases (P)	3		Extract with alcohol	O. Ad., Ext.
		Immunodeficiency (C)	3		Extract with alcohol	O. Ad., one tablespoon every day
		Circulatory disorders (C)	1		Extract with alcohol	O. Ad., one tablespoon every day
	*Scorzonera hispanica* L. (MFMTR26)	Gastric and intestine diseases (C)	3	Roots	Decoction	O. Ad.
	*Achillea millefolium* L. (MFMTR07)	Menorrhagia (C)	13	Aerial parts	Tea, extract with alcohol	O. Ad., drink extract 2–3 times per day, during menstruation
		Epistaxis (C)	8		Extract with alcohol	O. Ad.
		Kidney diseases (C)	2		Extract with alcohol	O. Ad.
	*Calendula officinalis* L.	Respiratory tract disorders (C)	8	Flowers	Tea	Inhalation; Ext., rinsing
		Inflammatory diseases (C)	7		Oil, concentrate, tea, extract with alcohol	O. Ad.
		Gastric and intestine diseases (C)	5		Tincture, tea, decoction	O. Ad.
		Menorrhagia, dysmenorrhea (C)	2		Tea, decoction	O. Ad.
		Purulent wounds (C)	6		Decoction	Ext., rinsing
	*Centaurea cyanus* L. (MFMTR45)	Cold, respiratory tract disorders (C)	2	Flowers	Extract with alcohol	O. Ad.
		Kidney and urinary tract diseases (C)	4		Extract with alcohol	O. Ad.
		Edema (P)	1		Extract with alcohol	O. Ad.
	*Helichrysum arenarium* (L.) Moench. (MFMTR28)	Vermin diseases (P)	1	Flowers	Decoction	O. Ad.
		Hepatic diseases and biliary problems (C)	1			O. Ad.
	*Matricaria recutita* L.	Insomnia, nervousness (C)	10	Flowers	Tea, decoction	O. Ad.
		Indigestion (C)	8			O. Ad.
		Cold, fever (C)	8			O. Ad.
	*Artemisia absinthium* L. (MFMTR42)	Indigestion (C)	4	Aerial parts	Tea, extract with alcohol	O. Ad.
		Insomnia, nervousness (C)	2			O. Ad.
		Vermin diseases (P)	1		Decoction	Ext., head rinsing
	*Artemisia vulgaris* L. (MFMTR17)	Indigestion (C)	6	Aerial parts	Tea, extract with alcohol	O. Ad.
		Insomnia, nervousness (C)	3			O. Ad.
	*Tussilago farfara* L. (MFMTR02)	Respiratory tract disorders (C)	5	Flowers, stem	Tea	O. Ad.
		Cold, fever (C)	8			O. Ad.
	*Arnica montana* L.	Joint pain (C)	3	Flowers	Extract with alcohol	Ext.
		Bruises, swellings (C)	1			Ext.
	*Arctium lappa* L. (MFMTR04)	Skin diseases (C)	3	Roots	Decoction	Ext.
		Indigestion and appetite loss (P)	2			O. Ad.
		Angina (C)	2			Ext., rinse throat
		Bruises, swellings (P)	1	Leaves	Clean leaves	Ext.
	*Taraxacum officinale* F. H. Wigg.	Indigestion and appetite loss (C)	5	Roots	Decoction	O. Ad.
		Purulent wounds (C)	2			Ext., rinsing
Berberidaceae	*Berberis vulgaris* L.	Gastric and intestine diseases (C)	1	Flowers, leaves, bark	Tea, extract with alcohol, decoction	O. Ad.
		Inflammatory diseases (P)	1		Extract with alcohol, decoction	O. Ad.
Betulaceae	*Betula pendula* Roth. (MFMTR22)	Indigestion and appetite loss (C)	1	Sap	Sap	O. Ad.
		Gastric acid hypersecretion (C)	3			O. Ad.
		Immunodeficiency (C)	2			O. Ad.
		Kidney and urinary tract diseases (C)	1	Leaves	Extract with alcohol	O. Ad.
	*Alnus incana* (L.) Moench. (MFMTR03)	Diarrhea (C)	1	Cones, bark, leaves	Decoction	O. Ad.
		Gastric and intestine diseases (P)	1		Extract with alcohol	O. Ad.
	*Corylus avellana* L. (MFMTR08)	Circulatory disorders (P)	1	Leaves	Tea, extract with alcohol, decoction	O. Ad.
Boraginaceae	*Myosotis arvensis* (L.) Hill. (MFMTR10)	Respiratory tract disorders (P)	2	Aerial parts	Extract with alcohol, decoction	O. Ad.
	*Symphytum officinale* L. (MFMTR12)	Respiratory tract disorders (P)	1	Roots	Extract with alcohol, decoction	O. Ad.
		Gastric and intestine diseases (P)	1			O. Ad.
		Bruises, swellings (C)	2		Compress	Ext.
		Joint pain (C)	2		Ointment	Ext.
Brassicaceae	*Lunaria annua* L.	Kidney and urinary tract diseases (C)	3	Aerial parts	Tea, decoction	O. Ad.
	*Eruca sativa* (Mill.) Thell.	Indigestion and appetite loss (C)	3	Aerial parts	Eat	O. Ad.
	*Armoracia rusticana* P. Gaertn., B. Mey et Scherb.	Inflammatory diseases (C)	3	Roots, leaves	Eat, juice	O. Ad.
		Indigestion and appetite loss (C)	8			O. Ad.
	*Capsella bursa-pastoris* (L.) Medik.	Hemorrhage (C)	2	Aerial parts	Tea	O. Ad.
		Kidney and urinary tract diseases (P)	1			O. Ad.
Cannabaceae	*Humulus lupulus* L.	Insomnia, nervousness (C)	6	Cones	Tea, extract with alcohol	O. Ad.
		Indigestion and appetite loss (C)	2			O. Ad.
Caprifoliaceae	*Viburnum opulus* L. (MFMTR25)	Menorrhagia (C)	2	Flowers, fruits, bark	Extract with alcohol	O. Ad., one tablespoon 2–3 times per day
		Gingivitis (C)	1			Ext., rinse mouth
		Immunodeficiency (P)	1		Extract with alcohol	O. Ad.
	*Valeriana officinalis* L.	Insomnia, nervousness (C)	25	Aerial parts	Tea, extract with alcohol	O. Ad.
Crassulaceae	*Rhodiola rosea* L.	Male potency disorders (C)	1	Roots	Decoction	O. Ad.
		Immunodeficiency (C)	1		Extract with alcohol, decoction	O. Ad.
Cucurbitaceae	*Cucurbita pepo* L.	Hypercholesterolaemia (C)	5	Seeds	Eat	O. Ad.
		Benign prostatic hyperplasia (C)	3		Eat	O. Ad.
Cupressaceae	*Juniperus communis* L. (MFMTR37)	Respiratory tract disorders (C)	4	Fruits	Tea, extract with alcohol	O. Ad.
		Kidney and urinary tract diseases (C)	3			O. Ad.
Dryopteridaceae	*Dryopteris filix-mas* (L.) Schott. (MFMTR40)	Vermin diseases (P)	3	Roots	Decoction	O. Ad.
		Gum disease (P)	2			Ext., rinse mouth
Elaeagnaoceae	*Hippophae rhamnoides* L.	Immunodeficiency (C)	2	Fruits	Juice, oil, extract with alcohol	O. Ad.
		Skin diseases (C)	4			O. Ad., Ext.
		Circulatory disorders (C)	1			O. Ad.
Equisetaceae	*Equisetum arvense* L. (MFMTR18)	Kidney and urinary tract diseases (C)	2	Aerial parts	Tea, extract with alcohol, decoction	O. Ad.
		Angina (P)	1			Ext., rinse throat
		Cardiovascular system disorders (P)	1			O. Ad.
Ericaceae	*Ledum palustre* L. (MFMTR58)	Respiratory tract disorders (C)	2	Shoot (1 year)	Decoction	O. Ad.
	*Arctostaphylos uva-ursi* (L.) Spreng. (MFMTR13)	Kidney and urinary tract diseases (C)	4	Leaves	Extract with alcohol, decoction	O. Ad.
	*Vaccinium uliginosum* L.	Indigestion (C)	3	Fruits, leaves	Eat, extract with alcohol	O. Ad.
		Kidney and urinary tract diseases (C)	2		Decoction	O. Ad.
	*Vaccinium myrtillus* L.	Diarrhea (C)	10	Fruits, leaves	Tea, extract with alcohol, decoction	O. Ad.
		Immunodeficiency (C)	5		Eat	O. Ad.
		Gastric and intestine diseases (C)	6			O. Ad.
	*Vaccinium vitis–idaea* L.	Kidney and urinary tract diseases (C)	14	Fruits, leaves	Tea, extract with alcohol, decoction	O. Ad.
		Immunodeficiency (C)	6		Eat	O. Ad.
	*Oxycoccus palustris* Pers.	Kidney and urinary tract diseases (C)	6	Fruits, leaves	Tea, extract with alcohol, decoction	O. Ad.
		Circulatory disorders (C)	2			O. Ad.
		Immunodeficiency (C)	3		Eat	O. Ad.
	*Calluna vulgaris* (L.) Hull. (MFMTR34)	Respiratory tract disorders (C)	2	Flowers, aerial parts	Tea, decoction	O. Ad.
		Cold, fever (C)	2			O. Ad.
		Insomnia, nervousness (P)	4			O. Ad.
Euphorbiaceae	*Euphorbia helioscopia* L. (MFMTR59)	Warts (C)	3	Stem	Stem	Ext. (lubricate on warts)
Fabaceae	*Ononis arvensis* L. (MFMTR36)	Cardiovascular system disorders (P)	1	Roots	Decoction	O. Ad.
		Kidney and urinary tract diseases (P)	1			O. Ad.
	*Trifolium pratense* L.	Respiratory tract disorders (C)	2	Flowers	Tea, decoction	O. Ad.
		Skin diseases (C)	3			O. Ad., Ext.
		Menopausal symptoms (C)	3			O. Ad.
Fagaceae	*Quercus robur* L. (12)	Diarrhea (C)	4	Bark, acorns	Coffee, tea, extract with alcohol, decoction	O. Ad.
		Indigestion and appetite loss (C)	7			O. Ad.
		Gum disease (C)	2			Ext., rinse mouth
Gentianaceae	*Centaurium erythraea* Rafn. (MFMTR15)	Indigestion and appetite loss (C)	4	Aerial parts	Tea, extract with alcohol	O. Ad.
		Constipation (C)	3			O. Ad.
Geraniaceae	*Pelargonium odoratissimum* (L.) L’Her.	Respiratory tract disorders (C)	1	Aerial parts		Inhalation
		Insomnia, nervousness (C)	1			Inhalation
Grossulariaceae	*Ribes uva-crispa* L.	Kidney and urinary tract diseases (C)	7	Fruits	Eat, tea	O. Ad.
	*Ribes nigrum* L.	Kidney and urinary tract diseases (C)	8	Fruits, leaves	Tea, extract with alcohol, decoction	O. Ad.
		Inflammatory diseases (P)	2			O. Ad.
		Vitiligo (C)	1			O. Ad., Ext.
		Immunodeficiency (C)	5			O. Ad.
Hydrangeaceae	*Philadelphus coronarius* L.	Circulatory disorders (P)	1	Flowers	Tea	O. Ad.
		Insomnia, nervousness (C)	2			O. Ad.
Hypericaceae	*Hypericum perforatum* L. (MFMTR39)	Kidney and urinary tract diseases (P)	2	Aerial parts	Tea, extract with alcohol, decoction	O. Ad.
		Skin diseases (C)	2			O. Ad., Ext.
		Insomnia, nervousness (C)	20			O. Ad.
		Gastric and intestine diseases (C)	4			O. Ad.
Lamiaceae	*Ajuga reptans* L. (MFMTR38)	Menorrhagia (C)	2	Aerial parts	Tea	O. Ad.
	*Mentha arvensis* L.	Cold, fever (C)	3	Leaves, aerial parts	Tea, extract with alcohol	O. Ad.
		Respiratory tract disorders (C)	3			Inhalation
		Indigestion and appetite loss (C)	2		Tea, extract with alcohol	O. Ad., one tablespoon 30 min before meal
	*Mentha x piperita* L.	Cold, fever (C)	17	Leaves, aerial parts	Tea, extract with alcohol	O. Ad.
		Respiratory tract disorders (C)	13			O. Ad.; Inhalation
		Indigestion and appetite loss (C)	5			O. Ad.
		Gastric and intestine diseases (C)	2			O. Ad.
		Circulatory disorders (P)	2			O. Ad.
		Insomnia, nervousness (C)	6			O. Ad.
		Inflammatory diseases (C)	3			O. Ad.
	*Origanum vulgare* L.	Insomnia, nervousness (C)	1	Aerial parts	Tea, extract with alcohol	O. Ad.
		Respiratory tract disorders (C)	3			O. Ad.
		Indigestion and appetite loss (C)	3			O. Ad.
		Joint pain (C)	1		Compress	Ext.
	*Thymus serpyllum* L.	Respiratory tract disorders (C)	6	Aerial parts	Tea, extract with alcohol, decoction	O. Ad.; Inhalation
		Insomnia, nervousness (C)	2			O. Ad.
		Gum disease (C)	1			Ext., rinse mouth
	*Thymus vulgaris* L. (MFMTR41)	Respiratory tract disorders (C)	13	Aerial parts	Tea	O. Ad.; Inhalation
		Insomnia, nervousness (C)	4			O. Ad.
	*Marrubium vulgare* L. (MFMTR48)	Hepatic diseases and biliary problems (P)	1	Leaves, aerial parts	Tea, extract with alcohol, decoction	O. Ad.
		Kidney and urinary tract diseases (C)	1			O. Ad.
		Respiratory tract disorders (C)	2			O. Ad.
	*Stachys officinalis* L. Trevis. (MFMTR43)	Insomnia, nervousness (C)	5	Leaves, aerial parts	Tea, extract with alcohol	O. Ad.
		Respiratory tract disorders (C)	8			O. Ad.
	*Lamium album* L. (MFMTR47)	Kidney and urinary tract diseases (P)	2	Leaves, aerial parts	Tea, extract with alcohol, extract with hot water	O. Ad.
		Respiratory tract disorders (C)	14			O. Ad.
		Cold, fever (C)	8			O. Ad.
		Menorrhagia (C)	2			O. Ad.
		Insomnia, nervousness (C)	4		Extract with alcohol	O. Ad
		Oncological diseases (C)	1			O. Ad
	*Salvia officinalis* L.	Respiratory tract disorders (C)	10	Leaves, aerial parts	Tea, extract with alcohol	O. Ad.; Inhalation
		Gum disease (C)	2			O. Ad.
		Insomnia, nervousness (C)	2			O. Ad.
	*Melissa officinalis* L.	Insomnia, nervousness (C)	20	Leaves, aerial parts	Tea	O. Ad.
		Menorrhagia (C)	4			O. Ad.
		Dysmenorrhea (C)	4			O. Ad.
	*Hyssopus officinalis* L.	Wounds (P)	1	Aerial parts	Extract with alcohol	Ext., rinsing
		Nervousness, anxiety (P)	1			O. Ad.
Linaceae	*Linum usitatissimum* L.	Constipation (C)	5	Seeds	Tea, decoction	O. Ad.
		Gastric and intestine diseases (C)	2			O. Ad.
Lycopodiaceae	*Lycopodium clavatum* L. (MFMTR14)	Respiratory tract disorders (C)	1	Spores		
		Kidney and urinary tract diseases (C)	2			
		Skin diseases (C)	2			Ext.
Malvaceae	*Tilia cordata* Mill.	Respiratory tract disorders (C)	22	Leaves, flowers	Tea	O. Ad.
		Insomnia, nervousness (C)	5			O. Ad.
		Cold, fever (C)	20			O. Ad.
		Kidney and urinary tract diseases (C)	5			O. Ad.
Menyanthaceae	*Menyanthes trifoliata* L. (MFMTR49)	Indigestion and appetite loss (C)	2	Leaves	Tea, extract with alcohol	O. Ad.
		Joint pain (P)	1		Compress	Ext.
Oleaceae	*Fraxinus excelsior* L.	Kidney and urinary tract diseases (P)	1	Leaves, bark	Tea, extract with alcohol	O. Ad.
		Joint pain (C)	1		Compress	Ext.
	*Syringa vulgaris* L.	Joint pain (C)	1	Flowers, buds	Extract with alcohol, compress	Ext.
Oxalidaceae	*Oxalis acetosalla* L. (MFMTR44)	Immunodeficiency (P)	1	Leaves	Eat (at spring)	O. Ad.
		Scurvy (P)	1			O. Ad.
Paeoniaceae	*Paeonia officinalis* L. *‘Rubra Plena’*	Insomnia, nervousness (C)	4	Flowers	Tea, extract with alcohol	O. Ad.
Papaveraceae	*Chelidonium majus* L. (MFMTR46)	Hepatic diseases and biliary problems (C)	3	Aerial parts	Tea, extract with alcohol, decoction	O. Ad.
		Insomnia, nervousness (C)	3			O. Ad.
		Oncological diseases (C)	2			O. Ad.
		Respiratory tract disorders (C)	6			O. Ad.
	*Papaver rhoeas* L.	Insomnia, nervousness (C)	4	Petals	Tea, extract with alcohol	O. Ad.
Parmeliaceae	*Cetraria islandica* (L.) Ach.	Respiratory tract disorders (C)	3	Wisps	Tea, extract with alcohol, decoction	O. Ad.
		Skin diseases, burns (P)	1		Decoction	Ext., rinsing
Pinaceae	*Picea abies* (L.) H. Karst.	Respiratory tract disorders (C)	6	Barbs, buds	Tea, extract with alcohol, decoction	O. Ad.; Inhalation
	*Pinus sylvestris* L.	Respiratory tract disorders (C)	8	Barbs, buds	Tea, extract with alcohol, decoction	O. Ad.; Inhalation
		Insomnia, nervousness (C)	1			O. Ad.
Plantaginaceae	*Plantago major* L. (MFMTR27)	Gastric and intestine diseases (C)	3	Leaves, seeds	Tea	O. Ad.
		Constipation (C)	4			O. Ad.
		Bruises, swellings (C)	4		Clean leaves	Ext. (put on bruises, swellings)
Poaceae	*Hierochloe odorata* (L.) P. Beauv. (MFMTR50)	Indigestion and appetite loss (P)	1	Leaves	Extract with alcohol	O. Ad.
	*Avena sativa* L.	Insomnia, nervousness (P)	2	Aerial parts	Extract with alcohol	O. Ad.
	*Elytrigia repens* (L.) Nevski. (MFMTR51)	Kidney and urinary tract diseases (P)	1	Aerial parts, rhizomes	Tea	O. Ad.
		Indigestion (C)	2		Eat	O. Ad.
	*Secale cereale* L.	Cardiovascular system disorders (C)	2	Grains	Extract with alcohol	O. Ad.
Polygonaceae	*Rheum rhabarbarum* L.	Indigestion (C)	3	Leaf stalks	Eat, juice, tea	O. Ad.
		Constipation (C)	4	Roots	Decoction	O. Ad.
	*Bistorta major* Gray. (MFMTR21)	Gastric and intestine diseases (C)	2	Rhizomes	Decoction	O. Ad.
		Kidney and urinary tract diseases (P)	1			O. Ad.
		Oncological diseases (C)	1			O. Ad.
		Gum disease (C)	1			Ext., rinse mouth
	*Polygonum aviculare* L. (MFMTR23)	Hepatic diseases and biliary problems (C)	3	Aerial parts	Tea, extract with alcohol, decoction	O. Ad.
		Hemorrhage (C)	2			O. Ad.
		Respiratory tract disorders (C)	2			O. Ad.
	*Persicaria maculosa* Gray. (MFMTR56)	Hemorrhage (P)	2	Aerial parts	Tea, extract with alcohol	O. Ad.
		Constipation (C)	1			O. Ad.
Primulaceae	*Primula veris* L. (MFMTR53)	Respiratory tract disorders (C)	5	Roots, flowers	Extract with alcohol, decoction	O. Ad.
		Migraine (C)	1			O. Ad.
Ranunculaceae	*Pulsatilla pratensis* (L.) Mill. (MFMTR54)	Insomnia, nervousness (C)	2	Aerial parts	Tea	O. Ad.
		Menorrhagia, dysmenorrhea (C)	2			O. Ad.
Rhamnaceae	*Frangula alnus* Mill.	Constipation (C)	3	Bark	Tea, decoction	O. Ad.
		Poisoning (P)	2			O. Ad.
		Gout (C)	1			O. Ad.
Rosaceae	*Filipendula ulmaria* (L.) Maxim. (MFMTR60)	Cold, fever (C)	4	Flowers	Tea, extract with alcohol	O. Ad.
		Kidney and urinary tract diseases (C)	2			O. Ad.
	*Rubus idaeus* L.	Cold, fever (C)	26	Berries, leaves	Tea	O. Ad.
		Skin diseases (C)	4			O. Ad., Ext.
	*Rosa canina* L.	Circulatory disorders (C)	5	Fruits	Tea, extract with alcohol	O. Ad.
		Anemia (C)	4			O. Ad.
		Immunodeficiency (C)	2			O. Ad.
	*Potentilla erecta* (L.) Raeusch. (MFMTR24)	Gastric and intestine diseases (C)	4	Rhizomes	Decoction	O. Ad.
		Gum disease (C)	2			Ext., rinse mouth
		Hemorrhage (C)	3			O. Ad.
	*Fragaria vesca* L.	Cold, fever (C)	12	Berries, leaves	Tea, extract with alcohol, syrup	O. Ad.
		Immunodeficiency (C)	3			O. Ad.
		Kidney and urinary tract diseases (C)	2			O. Ad.
	*Alchemilla vulgaris* L. (MFMTR19)	Gynecological diseases (C)	4	Aerial parts	Tea, extract with alcohol	O. Ad.
		Insomnia, nervousness (P)	2			O. Ad.
		Hemorrhage (C)	4			O. Ad.
	*Sorbus aucuparia* L.	Indigestion (C)	5	Fruits, bark	Tea, extract with alcohol, decoction, syrup	O. Ad.
		Kidney and urinary tract diseases (C)	4			O. Ad.
		Immunodeficiency (C)	3			O. Ad.
		Menopause (C)	8			O. Ad.
		Oncological diseases (C)	3			O. Ad.
	*Crataegus rhipidophylla* Gand.	Cardiovascular system disorders (C)	5	Berries, leaves	Tea, extract with alcohol	O. Ad.
		Insomnia, nervousness (C)	2			O. Ad.
	*Padus avium* Mill.	Wounds (C)	2	Berries, bark	Extract with alcohol, decoction	Ext., rinsing
		Skin diseases (C)	3			Ext., rinsing
Rubiaceae	*Galium verum* L. (MFMTR30)	Kidney and urinary tract diseases (C)	1	Flowers, herb	Tea, extract with alcohol	O. Ad.
		Wounds (P)	1			Ext., rinsing
Rutaceae	*Ruta graveolens* L.	Hemorrhage (P)	3	Aerial parts	Extract with alcohol, decoction	O. Ad.
		Snake bite (P)	1			Ext.
		Gynecological diseases (C)	3			O. Ad.
Santalaceae	*Viscum album* L.	Cardiovascular system disorders (P)	2	Leaves, twigs	Extract with alcohol, decoction	O. Ad.
		Oncological diseases (C)	2			O. Ad.
Sapindaceae	*Acer platanoides* L.	Indigestion and appetite loss (C)	3	Sap	Sap	O. Ad.
	*Aesculus hippocastanum* L.	Circulatory disorders (C)	4	Flowers, fruits	Tea, extract with alcohol	O. Ad.
Scrophulariaceae	*Verbascum spp.*	Oncological diseases (P)	1	Flowers	Extract with alcohol, extract with hot water	O. Ad.
		Insects (snakes) bites (P)	1			Ext.
	*Veronica officinalis* L. (MFMTR32)	Kidney and urinary tract diseases (P)	1	Aerial parts	Tea	O. Ad.
		Insomnia, nervousness (P)	1		Extract with alcohol	O. Ad.
		Allergies (P)	1		Tea	O. Ad.
	*Euphrasia rostkoviana* Hayne. (MFMTR57)	Eyes disease (C)	3	Aerial parts	Tea, compress	O. Ad., Ext.
Solanaceae	*Solanum tuberosum* L.	Gastric and intestine diseases (C)	2	Corms	Juice	O. Ad.
		Skin diseases (C)	1			O. Ad., Ext.
		Wounds (C)	1			Ext.
	*Solanum dulcamara* L. (MFMTR33)	Skin diseases (P)	1	Stem, bark	Extract with alcohol, decoction	Ext.
		Wounds (P)	1			Ext.
Sphagnaceae	*Sphagnum spp.*	Wounds (P)	1	Wisps	Wisps	Ext. (put on wounds)
		Inflammatory skin diseases (P)	1			Ext.
Urticaceae	*Urtica dioica* L.	Kidney and urinary tract diseases (C)	6	Leaves, aerial parts	Tea, extract with alcohol, decoction	O. Ad.
		Hemorrhage (C)	2			O. Ad.
		Menorrhagia (C)	2			O. Ad.
		Cold, fever (C)	5			O. Ad.
		Wounds (C)	2			Ext.
		Hair loss (C)	4			Ext., head rinsing
		Skin diseases (C)	2			O. Ad., Ext.
		Anemia (C)	1			O. Ad.
Violaceae	*Viola arvensis* Murray. (MFMTR29)	Kidney and urinary tract diseases (C)	3	Aerial parts	Tea, extract with alcohol	O. Ad.
		Skin diseases (C)	2			O. Ad., Ext.

*(C)* the current uses, *(P)* the past uses, *O. ad.* oral administration, *Ext.* external use

## Results and discussion

### Demographic data of the respondents

The respondents’ age varied between 50 and 92 years; 64.3% of all respondents were older than 65 years. We can hypothesize that respondents at this age use ethnopharmacological information they have learned before and get less information from mass media. Ninety percent of the respondents provided more than one source of information. Even 52% of respondents said that folk medicine was accumulated from their parents or grandparents. This shows that the collected ethnopharmacological material has been passed on from previous generations. It is equally important that 16% of respondents indicated learning about folk medicine from their neighbors and friends, since oral communication is the most common. Other researchers have highlighted that Samogitians are a closed community and usually knowledge of ethnomedicinal information is shared only with family members, so it was difficult to collect this information in the past [28]. Only 10% of respondents mentioned that information was obtained from radio, television, or internet sources and 3.4% referred to studies as another source. To assess the change in responses and collected samples, it was equally important to assess whether the respondents came from or lived in the investigated areas all the time. An absolute majority named Telšiai district as their place of residence. For the reasons mentioned above, we can assume that the majority of the collected ethnopharmaceutical material is of local use for the studied area.

Also, the respondents’ education was important for the study. While 78.6% of respondents had completed secondary education, 21.4% had not. These results demonstrate that in the twenty-first age, a quite high percent (21.4%) of respondents did not have a speciality and all respondents were farmers and housewives, who still had strong connections with traditional agricultural activities.

### Methods of collecting, preparing, and consuming medicinal plant materials

According to the study, respondents knew the best time to collect medicinal herbs for maximal effect. Most were guided by various calendar information and seasonality. Respondents knew that the best time to collect herbal raw materials is at dawn. Other researchers have assumed that the use of cultivated plants may increase as people have fewer encounters with wild plants. Wild plants are becoming more distant and less known as a result of habitat loss [4, 8]. In our study, the cultivated herbs make up to 25.6%, while 74.4% are harvested from the natural vegetation. It can be explained by a tradition to harvest herbal material from the wild and by the fact that all respondents had lived in the studied area all their life and knew natural plant growth sites very well.

Cultivated plants used for food, like garlic, onion, celery, potatoes, carrots, pumpkin, strawberries, beans, rhubarb, currants, and raspberries, are used as medicinal plants as well. It is usual for ethnobotany field studies to analyze wild plants used for both food and healing practices, because usually, these are interrelated areas [8, 29–31]. Oregano, parsley, poppy, sage, caraway, dill, horseradish, fennel, and thyme are also used both as spices and for medicinal purposes. Kasper-Pakosz and colleagues have done a study in the open-air markets of southeastern Poland and highlighted that species like oregano are relatively new to mainstream Polish cuisine and became fashionable only a few years ago. We noticed a similar tendency because Poland is a neighbor country and has similar history and cuisine traditions [5].

**Fig. 2:**
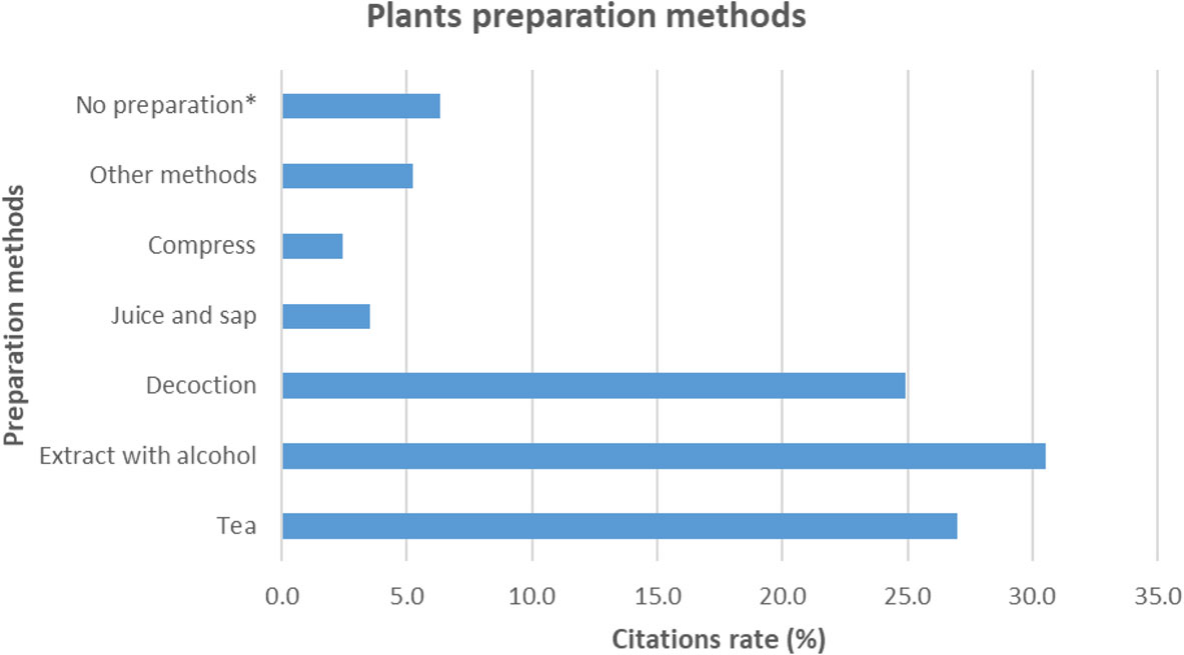
Preparation methods of herbal material

Respondents knew how to properly dry medicinal plant materials. In most cases, they spread them in a thin layer on newspapers or cotton material and put in a well-ventilated place until the herbs were dry. All dried herbs were stored in a dark, cool place, in paper or cotton bags, glass pots, or in paper boxes. Most respondents emphasized the importance of protecting herbs from direct sunlight and not leaving near electrical appliances, so they would not lose their healing properties. Respondents collected medicinal herbs to last 1 or 2 years. Some tended to pick fresh herbal raw materials every year to ensure the most effective herbal function. After their expiring time, they were burned, thrown away, or fed to animals. Some respondents used expired medicinal herbs for baths. The respondents also used ornamental plants for treatment. Respondents tended to use for treatment dried plant raw materials, but they also said they were using fresh and unprocessed material. After analyzing the data of the study, we distinguished the most frequently reported methods of preparation and use of medicinal plant raw materials. The most popular way to prepare herbs (30.5% of the total number of reports) was the extraction with alcohol (Fig. 2). Herbal raw materials prepared in this way can be stored for a long time and used as needed. Other popular methods were teas and decoctions, respectively 27% and 24.9%, because they are the fastest and most convenient forms of herbal preparations and do not take much time and additional stages. Interestingly, a large proportion of the respondents used medicinal herbs when they were still fresh—6.3% of the total number of reports. This method does not require almost any preparation and is effective and easy to apply. In this way, respondents usually used fruits and leaves. Other methods, such as preparation of extracts with hot water, oils, ointments, syrups, concentrates, and tinctures, make up 5.3% of the total number of reports.

### Materials of herbal origin used for medicinal purposes

We documented 125 species of medicinal plants from 55 families used for therapeutic purposes. Herbal raw materials are the most used material for medicinal purposes—91.2% of all registered material. The Asteraceae family had the highest number of reports being mentioned 147 times (16.6% of all reports). Other most popular families were as follows: Lamiaceae was mentioned 128 times, Rosaceae 108 times, Ericaceae 62 times, and Apiaceae 41 times. These plant families are dominant in other European ethnobotanical studies as well [7, 32]. Also, the high number of uses of Ericaceae family may be due to the intensive use of forest fruits, like it has been stated in studies done in neighboring countries [4, 8]. There are plant families, of which only one species is popular but is often mentioned; for example, one species of the Malvaceae family was reported 26 times.

During the study, it was important to find out what herbal raw materials respondents used for treatment and to systematize indications. According to the survey, the medicinal herb *Rubus idaeus* L. is the most popular species used by the respondents, local name aviete (Table 1). It was mentioned by respondents 28 times. Respondents used raspberry leaves and berries tea to cure a cold and fever and also to treat skin diseases, mostly by external use. In the second place, according to the report number, was a marigold (*Calendula officinalis* L.). Respondents mentioned it 27 times. Inhabitants of the Telšiai district used marigold flower tea, tinctures, oil, and extract with alcohol to treat respiratory tract or inflammatory diseases and gastric and intestine disorders. A decoction of marigold flowers was used by respondents to rinse purulent wounds. Women used marigold in composition with other herbs (melissa, camomile, white nettle) for menorrhagia and dysmenorrhea. In the third place were camomile (*Matricaria recutita* L.) and small linden tree (*Tilia cordata* Mill.) with the same number of reports. Inhabitants of the Telšiai district used camomile flower teas or decoctions from insomnia and nervousness, also for digestive tract disorders, a cold, and fever. A tea made of small linden tree flowers and leaves treats respiratory tract, kidney, and urinary tract disorders. Also, respondents used it for insomnia and nervousness, either when they get a cold or have a fever. Both these plants are popular in other European countries too. As recreational teas, *Matricaria chamomilla*, *Mentha spp*., *Rubus idaeus*, and *Tilia spp*. are widely used by Hutsuls living on both sides of the border between the Romanian and Ukrainian sides of Bukovina [33].

### Indications for the use of medicinal herbal raw materials

After detailed analysis of the research data, we separated the indications for treatment with herbal raw materials and grouped them from the most to the least mentioned.

**Fig. 3:**
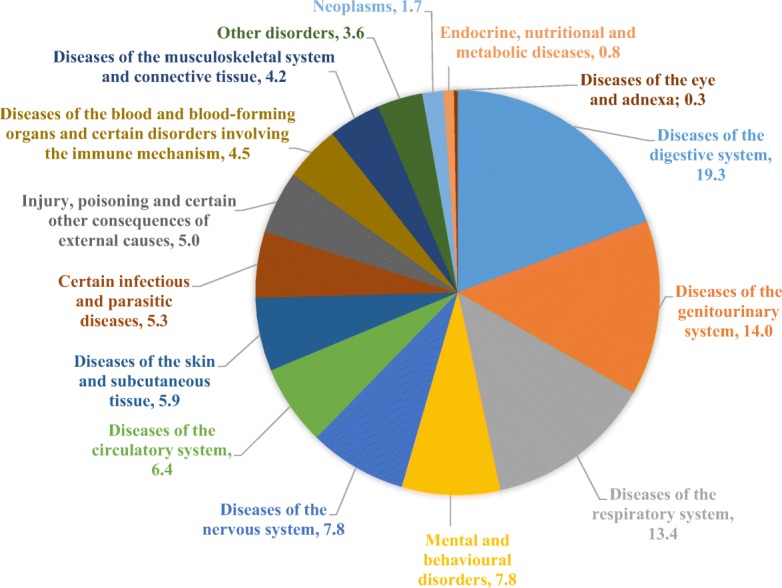
Indications for the use of medicinal herbal materials

Most often, respondents identified only certain symptoms of the disease, for example, a fever, pain, irritation, which were treated with medicinal herbal remedies. Also, it was common to explain the application of herbal raw materials for treatment in general terms, for example, improvement of digestive tract activity and treatment of respiratory diseases. The use of household and old names of diseases confirms that the information provided by these respondents was taken from the previous generations. In Table 1, we presented the ailments mentioned by respondents and medicinal plants used for their treatment. This information is important from the ethnopharmaceutical side. In Fig. 3, we presented all these ailments in general grouped into groups of diseases according to the international disease classification [34]. In this way, we could monitor the tendency for certain indications.

Figure 3 shows that most of the respondents in the investigated area used plant materials for treatment of digestive system (19% of the total number of reports). This can be explained by the abundant and calorific Lithuanian cuisine [35], which may lead to gastrointestinal disturbances. Also, a large proportion of herbal raw materials was used to treat genitourinary system diseases and ailments (14% of the total reports) and respiratory system diseases (13% of the total number of reports). Other two most popular indications may be influenced by cool and moist northern Lithuanian climate. Most of genitourinary system diseases treatments were gender-specific, such as treatments of dysmenorrhea, menorrhagia, or other gynecological disturbances. This fact has been noticed in review studies as well [36]. In Lithuanian villages, *women’s illnesses* used to be treated using folk medicine. Nowadays, it is still usual to treat mild disorders with homemade remedies (mainly of herbal origin). Also, up to 8% of all indications belong to nervous system and behavioral disorders. In this study, the most often mentioned disorders were *fear* and insomnia. Other unpublished studies done in Lithuania on ethnopsychiatry have highlighted that the cause of nervous system disorder most often is considered to be *fear*, strong emotional experiences, and fear of thunder, animal, or human [16, 37]. These specific diseases have always been in the villages but have not always been possible to treat with the help of proper medical care [37].

According to the survey, respondents tended to use herbal raw materials both in the treatment of specific diseases, whose names they knew precisely, as additional measures to reduce the use of chemical drugs, and for prevention, in order to avoid certain diseases or ailments.

### Parts of plants used for the treatment

We found that respondents knew that certain parts of the medicinal plant accumulate more nutrients and their action can be more effective. The most commonly used parts for treatment according to respondents are aerial parts—24% of all reports. This way of collecting and preparing raw materials is simple and does not take much time and effort. Also popular was the use of leaves (19%) and flowers (13% of the total number). Leaves and flowers are commonly used as a medicine in many parts of the world [38]. Researches have highlighted that the herbal material for medicinal purposes is commonly used because it is abundantly available and easy to harvest [33, 36]. Roots/rhizomes and fruits/berries were used similarly often, 12% and 11%, respectively. These raw materials are more difficult to collect and prepare for treatment, because they are hard and require additional preparation. In many cases, dried wood or roots can maintain bioactive compounds for longer time after harvesting than leaves or flowers [33], and according to interviewers “these parts are better for storing.” Other plant parts such as bark, seeds, buds, corms, and stems were used less than 5% of all plant parts mentioned in the study. Yet, other mentioned plant parts such as acorns, barbs, cones, grains, petals, sap, spores, twigs, and wisps made up 8%. According to interviewers, most often, these raw materials are collected and used when “they contain the most nutrients,” which requires knowledge and observation.

### Comparative analysis with prior researchers

In order to observe the trends in the use of folk medicine in Lithuania and to evaluate the range of ethnopharmaceutical knowledge, the data obtained during the study were compared with an earlier similar study in Lithuania. The chosen study is of similar nature, with an accurate analysis of medicinal plant families, which enabled us to successfully analyze the results obtained. In 2006–2007, we made our first attempt to collect and analyze ethnobotanical knowledge in Lithuania. The research was done in the same Samogitia region with strong cultural and historical background [6].

Before conducting a peer-to-peer analysis, it was expected that the use of herbal raw materials, fungi, and substances of animal origin used for medicinal purposes would decrease over time. This assumption was made by evaluating rapid industrialization and availability of chemical drugs in Lithuanian towns and villages.

Between these two researches in Samogitia region, there is a 10 years gap. The study accomplished in 2006–2007 interviewed 20 respondents, who referred to 119 medicinal plants from 56 plant families used for various medical indications. Meanwhile, in our 2016–2017 study, we interviewed 28 respondents from Telšiai region, who referred to 125 plants from 55 plant families used for medical purposes. Both studies have shown that the most collected plant families were Asteraceae and Lamiaceae. In these studies, the most used medicinal plants were a bit different: in the earlier study, the most popular were *Calendula officinalis* L. and *Vaccinium vitis-idaea* L.; meanwhile, in this study, the most widely used medicinal plants were *Rubus idaeus* L. and *Calendula officinalis* L. The most popular plant preparations for use in both studies were tea and with alcohol extract. In both studies, the most popular material of animal origin was *Bufo bufo*, and the most commonly used fungus was *Phallus impudicus*. The most popular mineral material in Samogitia traditional medicine was sand. In both studies, medicinal plants were the most frequently used for treatment of digestive tract disorders and disorders of respiratory tract. Comparative analysis with the previous survey in this region showed similar results and discovered a large amount of ethnopharmaceutical information.

Although the pharmaceutical industry is expanding rapidly, pharmacies are becoming more and more accessible, and the influence of advertising on chemical drugs, food supplements, and other preparations is quite large; the use of natural remedies and the application of folk medicine knowledge are still in the midst of residents of the Telšiai district. Nevertheless, the active treatment of medicinal natural substances may also be affected by the fact that many respondents live far away from larger cities, and due to their poor connectivity, they are rarely visited. Also, distrust of doctors and pharmacists on the part of the respondents means that they tend to rely on the healing properties of natural remedies.

In the twenties and thirties of the nineteenth century, Vilnius (the current capital of Lithuania) was a traditional center of harvesting and wholesale trade of medicinal plants [14]. A study done in 1993 found more than 100 food plants applied into Lithuanian ethnopharmacology. The most numerous and diverse plant species were utilized as gastro-intestinal remedies, diuretic or pulmonary aid, sedative, heart and vascular medicines, and dermatological aid [14]. Indications for treatment with herbal medicine in Lithuania compared with our study are very similar (Fig. 3). According to this study, we highlighted limitations in our work—we did not investigate the use of plants for medical and/or food purposes and this is an idea for future studies.

### Substances of animal origin used as medicines

In this study, it was not expected to capture a large number of animal-derived substances used for therapeutic purposes, as these substances have become less commonly used and unpopular in comparison with plant-based substances. Nine animal species were recorded during the study period. Three animal species were still used for medicinal purposes; the other six were mentioned only as historical medicines.

**Fig. 4:**
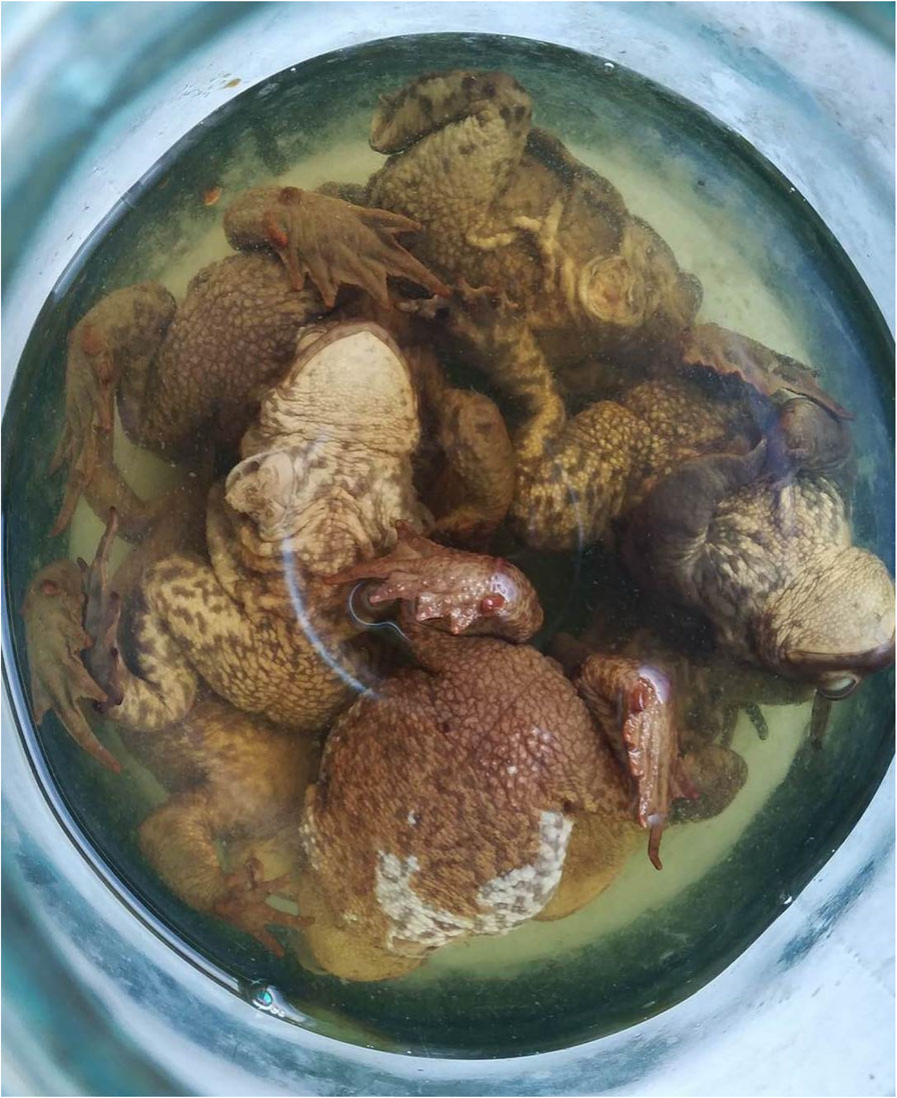
Material of animal origin used for medicinal purposes (extract with alcohol, toad)

The most common animal still used for medicinal purposes in the studied region was the toad (*Bufo bufo*) (Fig. 4) to compare with the first study in Samogitia, when the toad as well was the most popular of animal origin substances (89% and 95% of respondents, respectively) [39]. It was used to treat oncological diseases and for prophylaxis, as well as for stomach diseases or digestive disorders. All respondents highlighted the use of homemade alcohol to prepare homemade medicines. There are different ways to prepare the animal for the folk medicines.

#### Extraction with ethanol

Catch the toad and put in a bucket, cover it to avoid getting out, fill the bucket with water, keep it for a day to settle changing the water several times. Have a clean jar ready. Take the toad, put in the prepared jar with ethanol, and close it with a lid. After a few minutes, the toad will die. Keep for a couple of months because you have to get out the poison, then you can throw the toad away. It is to be used a teaspoon daily, preferably in the evening, and can be diluted with water or tea. In the case of an oncological illness, it is necessary to drink it in the autumn and spring. Use for 2 to 3 weeks.

#### Burning to ashes

A live toad should be taken, closed in a tin, placed on fire, and burned to ashes (one of the oldest forms of medicine). After that, the toad needs to be crushed to powder (ashes). Ashes are mixed with honey and used a teaspoon in the morning. The toad can also be prepared otherwise. Stick a live toad on a prong and leave to dry in the sun. Powdered toad can be used mixed with honey a teaspoon in the morning or the whole toad can be cut into pieces. A piece should be boiled in water for a few minutes. Appropriate for children for *scarlet fever*.

For the upper respiratory tract diseases, it is used in several ways: (1) put a toad in the boiling milk for a few minutes, strain the milk, and add honey. Give a teaspoon few times a day to patients with laryngitis, bronchitis, or other respiratory diseases; (2) put a toad in the furnace, burn to fine ash, and crush further to be really fine. Put the powder (ashes) into a jar and keep it to be used as needed. Use for various respiratory tract diseases, mixed with honey to eat or put in warmed milk or water to drink.

#### Application with a live toad

In the case of tonsillitis, toad was wrapped in gauze and placed over the throat. Respondents said that they had seen with their own eyes how the toad was “spilling,” and then had to be removed because it was no longer needed—the toad had done its job and the patient had improved. Toads were also used to treat viper (*Vipera berus*) bite: a toad was to be placed on the bite place immediately; the toad would swell up and die, and the swelling would disappear. Also a toad was wrapped in gauze and applied on purulent wounds and ulcers.

Another commonly used animal (7%) is the only poisonous reptile in Lithuania, common adder (*Vipera berus*) or common viper. Its bite usually is not deadly. The viper is prepared as extract with alcohol and used for various gastric diseases as well as oncological diseases. The respondent said that there were a lot of snakes in their place of residence since it was a peatland area, and his father used to catch these snakes by doctor’s order. They were specifically used to treat stomach cancer. When the factory opened nearby, the population of snakes dropped sharply. Other respondent said that he used this extract for disturbed digestion and loss of appetite. For external use, the extract with alcohol was rubbed into painful joints. This extract was also known to be used for cosmetic purposes. The respondent said that she had a very pimpled face in her youth. Mom told her to apply a snake extract with alcohol; after a few days, the skin was clean, and her face became smooth. Also, a snake was boiled with potatoes and given to the animals when they ate poorly.

All these indications and methods of use were mentioned in the first study; the ethnomedical uses of these two animals are characteristic for this region and are still widely used [39].

Another animal used is a pig (*Sus scrofa domesticus*) (4%). It is the most popular domestic animal in Lithuania used for food and also for medicinal purposes. A pig bladder was used for inflammation of the bladder. The pig was slaughtered; the bladder was cut off and suspended over the furnace to dry. When necessary, a piece of it was cut to prepare a tea, used until all gone. Prostate diseases were treated with a dried pig urinary bladder consumed with hot water. Pig bile was used to treat burns. Pig bile extract with alcohol was used for stomach ulcer. Pig fat was used as an ointment base or applied to frozen body parts. These days, this treatment is no longer used due to the unappealing odor.

Also, bee products were and still are very popular in Lithuania [10, 40]. In Lithuanian ethnomedicine, we can find recipes with bee products, and even whole bees themselves, for the treatment of various ailments. Bee products can be used alone or in combination with medicinal plants and substances of animal origin for synergistic effect, cumulative impact, or just better taste and administration form. A study of archive material from ethnographic expeditions dated from 1886 to 1992 in different parts of Lithuania concerning historical use of bees and bee products showed that the most popular indications were ointments with honey to treat abscesses and wounds [10]. According to respondents of our study, for poorly healing wounds, there was the following ointment produced: propolis was mixed with homemade butter, put in a water bath, then allowed to harden and spread on the wounds. Other respondents used propolis to treat stomach ulcers: put about 8–10 g of propolis in 80–100 ml 70% alcohol and leave for about 2 weeks in a dark place; add few drops to a glass of water and drink in the morning.

In these days, bee honey is still being used because of its beneficial properties. Few respondents use it in ointment compositions with homemade butter or pig fat. Other respondents use honey as lip balm, because it not only softens and protects lips, but also heals the splits or frostbite. Honey is also included in the composition of many folk recipes as an ingredient (with onion, garlic, pig fats, toad, etc.). Honey is the most popular bee product used until now, according to ethnomedicine studies in other countries [41–43].

This study shows that recipes with bee products are still used in Lithuania as well. Scientists in Lithuania use bee products and the ideas from archival sources for further analysis and creation of cosmetic products [44–48].

### Magical and ritual healing procedures

In addition to medicinal plants and animal products, respondents identified other measures that are/were used for treatment. These methods are presented by respondents as ancient and no longer used, but sometimes, they admitted still using them. Rituals and herbal remedies are connected not in chaotic way but according to some models, which could be explained by different treatment of various disorders. Fumigation is very specific way of treating illnesses and, because of big variety of herbs used in this practice, should be considered rational and not only magical. Magical actions are taken to strengthen botanical remedies [16]. As mentioned by other authors, remedies are used in “magical” healing procedures which are of particular importance to the treatment of psychosomatic illnesses [49, 50].

Fumigation with blessed juniper (*Juniperus communis*) is still very popular in this region. The previous study [39] and this study have both recorded common cases of juniper burned and all corners of the home fumigated to protect against all sorts of diseases, disasters, and thunder. This shows an interrelation of Christian and pagan traditions, because juniper is blessed in the church according to Christian traditions and used to protect one’s home from thunder by pagan traditions. As the thunder approaches, people feel fear and that fear is believed to be the devil itself hiding next to the man. It is also believed that the devil is hiding behind the anger and jealousy and that a good man does not get hit by lightning. Thus, home protection from thunder by burning blessed herbs should be regarded as an attempt to scare away not Perkūnas but the evil spirits and devil hiding in the house, and thus leave no cause for a disaster, which is attracted by negative human emotions [16, 37]. Herb of *Artemisia absinthium* L. also used to be put in the house, burned, and fumigated with, because it was thought to protect from evil spirits and against diseases. Samogitians used *Fraxinus excelsior* to treat diseases and to scare away evil spirits: the young *Fraxinus* stems were picked and squeezed and the drops were put in the ears for the ear inflammation. If a bad energy was felt in the house or an unwanted person had visited, then it was fumigated with young stems with leaves.

When the whole family was sick, goose feathers were burned to clean the air and home from the disease. In the past, a person who had lungworms was also told to breathe smoke of burned goose feathers.

## Conclusions

Based on the results of this study, we highlighted the possible reasons why, 10 years after the first study, the amount of ethnopharmaceutical knowledge and application in the region remained unchanged. According to Lithuanian State Medicines Control Agency from 2003 to 2017, the number of pharmacies in Lithuania is decreasing and is concentrated in big cities [51]. In small towns and villages, the number of pharmacies is decreasing; because of low profit, there are no pharmacists who want to work away from the city. Moreover, none of the participants in the study are seeking a family doctor or a pharmacist to obtain information on medicinal plants, their use, and other natural treatments as they do not trust them. Collected ethnopharmacological material has been passed on from previous generations, as the main source of ethnopharmaceutical information was family members. Also as modern medical assistance is quite expensive, self-medication with homemade medicines is still popular here.

It shows how important it is to collect and systematize this information as soon as possible; to save this information as traditional Lithuanian heritage and also use it for scientific investigations. Researchers in Lithuania are trying to create unique products from traditional plants based on their historical background, but suitable for today’s user needs. It is very important not to leave the collected ethnopharmacological knowledge in the archives but present it to the scientific community and younger generation and to adapt it for the modern lifestyle.
